# Apparent diffusion coefficient histogram in breast cancer brain metastases may predict their biological subtype and progression

**DOI:** 10.1038/s41598-018-28315-y

**Published:** 2018-07-02

**Authors:** Sung Jun Ahn, Mijin Park, Sungkyu Bang, Eunseo Cho, Sung Gwe Ahn, Sang Hyun Suh, Jong-Min Lee

**Affiliations:** 1 Department of Radiology, Gangnam Severance Hospital, Yonsei University, College of Medicine, Seoul, Korea; 2 Department of Biomedical Engineering, Hanyang University, Seoul, Korea; 3 Department of Surgery, Gangnam Severance Hospital, Yonsei University, College of Medicine, Seoul, Korea

## Abstract

Our aims for this study were to investigate the relationship between diffusion weighted image (DWI) parameters of brain metastases (BMs) and biological markers of breast cancer, and moreover, to assess whether DWI parameters accurately predict patient outcomes. DWI data for 34 patients with BMs from breast cancer were retrospectively reviewed. Apparent diffusion coefficient (ADC) histogram parameters were calculated from all measurable BMs. Two region of interest (ROI) methods are used for the analysis: from the largest BM or from all measurable BMs per one patient. ADC histogram parameters were compared between positive and negative groups depending on ER/PR and HER2 statuses. Overall survival analysis after BM (OSBM) and BM-specific progression-free survival (BMPFS) was analyzed with ADC parameters. Regardless of ROI methods, 25th percentile of ADC histogram was significantly lower in the ER/PR-positive group than in the ER/PR-negative group (P < 0.05). Using ROIs from all measurable BMs, Peak location, 50th percentile, 75th percentile, and mean value of ADC histogram were also significantly lower in the ER/PR-positive group than in the ER/PR-negative group (P < 0.05). However, there was no significant difference between HER2-postive and negative group. On univariate analysis, using ROIs from all measurable BMs, lower 25th percentile, 50th percentile and mean of ADC were significant predictors for poor BMPFS. ADC histogram analysis may have a prognostic value over ER/PR status as well as BMPFS.

## Introduction

Breast cancer is the most common cancer in women world-wide and constitutes the second-most frequent cause of brain metastases (BMs), which occur in 10–16% of patients^1–3^. The incidence of BMs has increased in recent years, which is likely because of the prolonged survival of patients who receive more efficient treatments, along with the availability of better imaging techniques that enable increased detection of BM^2,4^. Despite the advent of better systemic therapies, BMs are a major cause of morbidity and are associated with progressive neurologic deficits that reduce quality of life^5^.

Breast cancer can be divided into three biologic subtypes, based on biomarkers such as the estrogen receptor (ER), progesterone receptor (PR), and human epidermal growth receptor 2 (HER2). Each subtype exhibits a distinct prognostic significance^6,7^. The subgroups of patients with triple-negative and HER2-positive breast cancer are at high risk for the development of BMs^8,9^. The onset of BMs occurs earlier in triple-negative breast cancer than in other subtypes, and the overall survival rate in these cases is particularly poor^10^. In addition, it is crucial to consider ER/PR and HER2 statuses, both for prognosis and for understanding the different systemic therapies available; importantly, HER2-targeted therapy successfully improved the overall survival in patients with HER2-positive breast cancer^11^.

Diffusion-weighted magnetic resonance (MR) imaging (DWI) of the brain is based on differential diffusion rates or the Brownian motion of water. It is an essential technique for diagnosing acute infarction in the brain because of its ability to detect the cytotoxic edema that is caused by altered water diffusion, secondary to cellular damage. DWI is also widely used for the assessment of tumor pathology in the field of neuro-oncology^12^. Specifically, apparent diffusion coefficient (ADC) values derived from DWI have been shown to correlate with tumor cellularity, glioma grade, and treatment response^12–16^.

We hypothesized that DWI parameters might correlate with biomarkers and patient prognosis in breast cancer patients with BMs. Our aims for this study were two-fold: (1) to investigate the relationship between DWI parameters and the HER-2 and ER/PR statuses of breast cancer, and (2) to assess whether DWI parameters accurately predict patient outcomes.

## Materials and Methods

### Participants

We retrospectively reviewed data for breast cancer patients with BMs who underwent gadolinium-enhanced brain MRI from 2011 to 2017. A total of 94 patients were identified. Of these, 58 patients were excluded for the following reasons: (1) previous neurosurgery or brain radiation therapy (n = 15); (2) presence of other malignant disease (n = 7); (3) absence of the immunohistochemistry profile of breast cancer (n = 15); (4) absence of diffusion-weighted images (n = 21); (5) poor image quality (n = 2). A total of 34 patients remained after exclusion was completed. Immunohistochemistry was performed to evaluate the levels of ER, PR, and HER2 expression in primary breast cancer. Fluorescent *in situ* hybridization analysis of HER2 amplification was performed inimmunohistochemistry 2 + cases. The current study design and use of clinical data was approved by Gangnam severance hospital institutional review board (protocol # 3-2017-0175). All experiments were carried out in accordance with approved guidelines. The requirement to obtain informed consent was waived, and all data were fully anonymized. Overall survival analysis after BM (OSBM) was defined as the time from initial BM diagnosis to the time of death or last follow-up. BM-specific progression-free survival (BMPFS) was defined as the time from the initial BM diagnosis to the time of BM progression.

### Imaging

All patients were imaged with a 3 T clinical MR imaging device (Discovery MR750, GE Healthcare, Milwaukee, Wisconsin, USA). Our MR imaging protocol for BM included routine diffusion-weighted echo-planar sequences (TR/TE, 8000/65.6 ms; slice thickness/intersection gap, 4/1 mm; matrix size, 160 × 160; FOV, 240 × 240 mm; three directions; b-value = 0 and 1000 s/mm^2^), and T2-weighted fast-spin-echo sequences (repetition time/echo time (TR/TE), 5414/96 ms). After intravenous gadolinium-based contrast agent was administered at a dose of 0.1 mmol/kg body weight, axial fluid-attenuated inversion recovery sequences (TR/TE/inversion time (TI), 4000/80/2000 ms) and 3D T1 fast-spoiled gradient-recalled sequences (TR/TE, 8.2/3.2 ms; flip angle 12°; slice thickness, 1 mm; matrix size, 256 × 256; FOV, 220 × 220 mm) were taken sequentially. ADC values were automatically calculated by the operating console of the MR imaging device and were displayed as corresponding ADC maps.

### Image postprocessing and analysis

**Figure 1:**
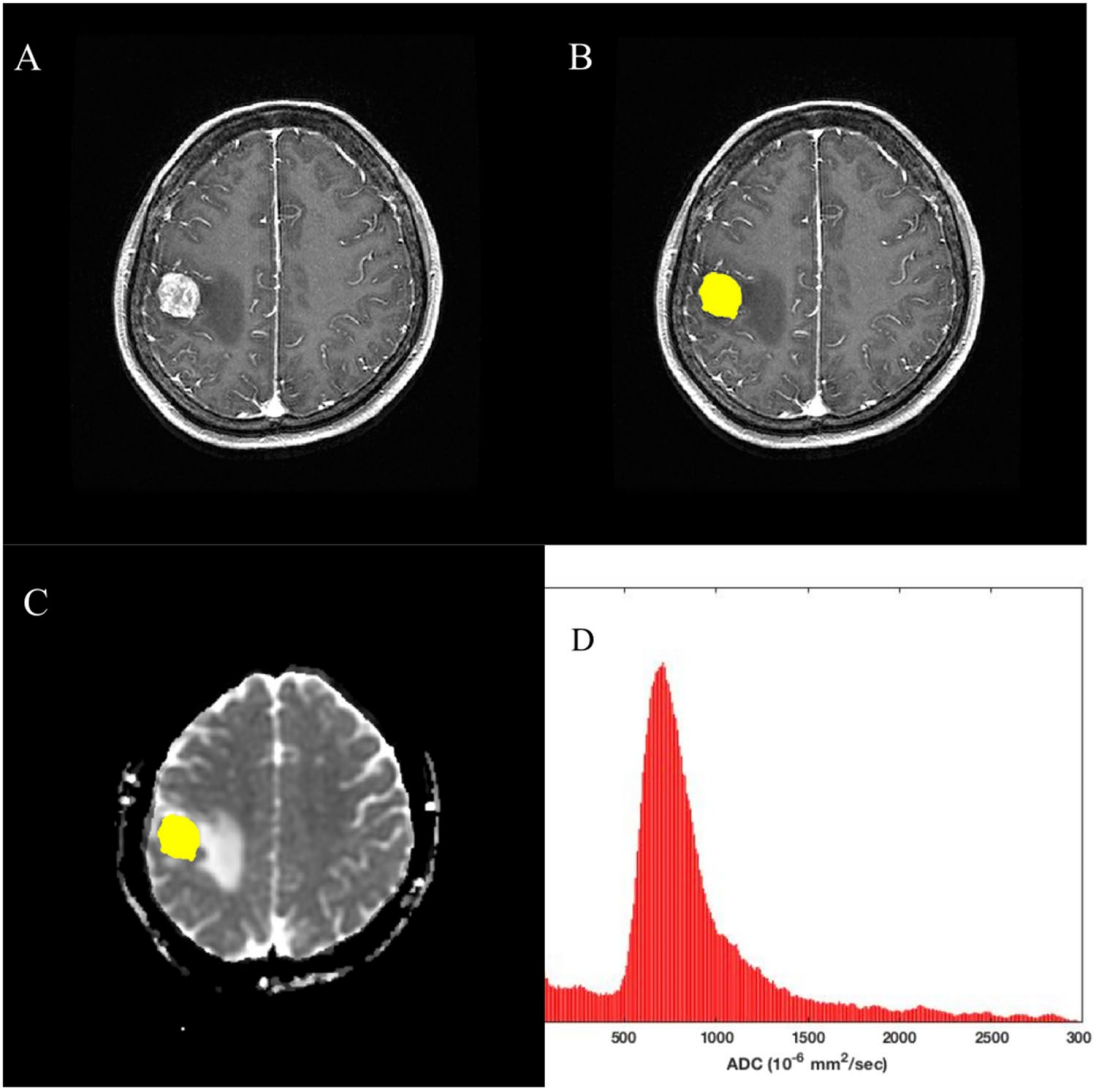
Processing workflow diagram. (**A**) contrast-enhanced T1-weighted MR image, (**B**) contrast-enhanced T1-weighted MR image with ROIs to segment the entire enhancing BM, (**C**) ADC map with the ROI of the entire BM overlaid, and (**D**) ADC histogram extracted from ROIs.

We regarded a BM as measurable when its volume is more than 100 mm^3^, because with the volume of less than 100 mm^3^, it is difficult to draw ROIs exactly and co register to ADC space correctly. Regions of interest (ROIs) were drawn on each tumor section on contrast-enhanced T1-weighted images using a free open-source toolkit, ITK-SNAP (www.itksnap.org)^17^. ROI drawings were not performed for non-measurable BMs. The ROI masks were automatically segmented with intensity thresholds; incomplete regions of the entire enhancing tumor were manually corrected. ROIs were co-registered to ADC maps via affine transformation with normalized mutual information as a cost function^18^. ADC histograms were generated with a bin of 1 × 10^−5^ mm^2^/sec and a range of 10–3000 × 10^−6^ mm^2^/sec. We considered ADC values <10 × 10^−6^ mm^2^/sec to be artifacts and values >3000 × 10^−6^ mm^2^/sec to be cystic portions. ADC histogram parameters (peak location, 25th and 75th percentile values, median, mean, and standard deviation) were calculated from ROIs that were overlaid on ADC maps. A representative case is presented in Fig. 1.

### Statistical analysis

Overall, we used two ROI methods for our study: First one is to use a ROI of largest BM from one patients. In this case, we used a total of 34 ROIs for analysis. Second one is to use ROIs from all measurable BMs. In this case, we used a total of 85 ROIs for analysis. ADC histogram parameters were compared between positive and negative groups depending on ER/PR and HER2 statuses, using a two-sample t-test. ADC variables were dichotomized into two groups by median and univariate survival analyses were performed to identify ADC variables to stratify OSBM and BMPFS, using a log-rank test.

## Results

### Patient characteristics

Patient characteristics are summarized in Table 1. No significant differences were found in clinical characteristics between groups (HER2-positive vs negative, ER/PR-postive vs negative). Mean ages at the initial diagnosis were 46.4 ± 12.8 years vs 46.6 ± 11.1 years (HER2-negative vs HER2-positive, p = 0.96), 45.3 ± 9.9 years vs 48.2 ± 14 years (ER/PR-negative vs ENR/PR positive, p = 0.51). The majority of tumor histologic type was ductal carcinoma. All BM patients underwent at least one treatment (surgery, radiation or systemic therapy). Volumes and diameters of BMs were not significantly different between groups. Mean diameters of largest BM were 19.9 ± 9.1 mm vs 20.3 ± 7.5 mm (HER2-negative vs positive, p = 0.86) and 18.5 ± 7.9 mm vs 22.0 ± 8.4 mm (ER/PR negative vs positive, p = 0.21). Mean volumes of largest BM were 6895 ± 10024 mm^3^ vs 6044 ± 5123 mm^3^ (HER2-negative vs positive, p = 0.86) and 5132 ± 6325 mm^3^ vs 7920 ± 8994 mm^3^ (ER/PR negative vs positive, p = 0.3). The number of measurable BMs were 85. Mean diameters of measurable BMs were 14.1 ± 7.2 mm vs 15.6 ± 7.4 mm (HER2-negative vs positive, p = 0.34) and 14.3 ± 6.7 mm vs 15.4 ± 7.8 mm (ER/PR-negative vs positive, p = 0.49). Mean volumes of measurable BMs were 2962 ± 6435 mm^3^ vs 3423 ± 4352 mm^3^ (HER2-negative vs positive, p = 0.69) and 2717 ± 4557 mm^3^ vs 3629 ± 6463 mm^3^ (ER/PR negative vs positive, p = 0.45). The mean number of measurable BMs per patient were 2.9 ± 2.9 vs 2.1 ± 2.0 (HER2-negative vs positive, p = 0.33) and 2.4 ± 2.4 vs 2.6 ± 2.6 (ER/PR negative vs positive, p = 0.78).

**Table 1:** Characteristics of breast cancer patients with brain metastases.

	HER2-negative (n = 16)	HER2-positive (n = 18)	P-value	ER/PR-negative (n = 18)	ER/PR-positive (n = 16)	P-value
Age at initial diagnosis, years	46.4 ± 12.8	46.6 ± 11.1	0.96	45.3 ± 9.9	48.2 ± 14.0	0.5
Initial TNM stage			0.66			0.09
I	1 (6.2%)	3 (16.7%)		3 (16.7%)	1 (6.2%)	
II	8 (50.0%)	6 (33.3%)		5 (27.8%)	9 (56.2%)	
III	4 (25.0%)	6 (33.3%)		8 (44.4%)	2 (12.5%)	
IV	3 (18.8%)	3 (16.7%)		2 (11.1%)	4 (25.0%)	
Histology			0.17			0.15
Ductal carcinoma	15 (93.8%)	14 (77.8%)		16 (88.9%)	13 (81.2%)	
Lobular carcinoma	0 (0.0%)	1 (5.6%)		1 (5.6%)	0 (0.0%)	
Metaplastic carcinoma	1 (6.2%)	0 (0.0%)		1 (5.6%)	0 (0.0%)	
Unknown	0 (0.0%)	3 (16.7%)		0 (0.0%)	3 (18.8%)	
Systemic therapy after BM			0.13			0.77
Yes	11 (68.8%)	17 (94.4%)		14 (77.8%)	14 (87.5%)	
No	5 (31.2%)	1 (5.6%)		4 (22.2%)	2 (12.5%)	
Surgery after BM			0.73			0.73
Yes	7 (43.8%)	10 (55.6%)		8 (44.4%)	9 (56.2%)	
No	9 (56.2%)	8 (44.4%)		10 (55.6%)	7 (43.8%)	
Radiation therapy after BM			0.17			0.87
Yes	9 (56.2%)	15 (83.3%)		12 (66.7%)	12 (75.0%)	
No	7 (43.8%)	3 (16.7%)		6 (33.3%)	4 (25.0%)	
Largest BMs						
Diameter (mm)	19.9 ± 9.1	20.3 ± 7.5	0.86	18.5 ± 7.9	22.0 ± 8.4	0.21
Volume (mm^3^)	6895 ± 10024	6044 ± 5123	0.76	5132 ± 6325	7920 ± 8994	0.3
Measurable BMs						
Diameter (mm)	14.1 ± 7.2	15.6 ± 7.4	0.34	14.3 ± 6.7	15.4 ± 7.8	0.49
Volume (mm^3^)	2962 ± 6435	3423 ± 4352	0.69	2717 ± 4577	3629 ± 6463	0.45
Number of BMs per one patient	2.9 ± 2.9	2.1 ± 2.0	0.33	2.4 ± 2.4	2.6 ± 2.6	0.78

BM, brain metastasis; ER/PR, estrogen receptor/progesterone receptor; HER2, human epidermal growth receptor 2; TNM, tumor-nodes.

### Relationship between ADC histogram and biological subtype

Regardless of ROI methods, ADC variables were not significantly different between HER2-positive and -negative groups. However, between ER/PR positive and negative groups, there was significant differences. Using ROI from largest BMs, 25th percentile of ADC histogram was significantly lower in the ER/PR-positive group than in the ER/PR-negative group (840 ± 184 × 10^−6^ mm^2^ vs. 965 ± 133 × 10^−6^ mm^2^/sec, *p* < 0.05, Table 2). Using ROIs from all measurable BMs, Peak location, 25th percentile, 50th percentile, 75th percentile and mean of ADC histogram were significantly lower in the ER/PR-positive group than in the ER/PR-negative group (899 ± 228 × 10^−6^ mm^2^ vs. 1020 ± 275 × 10^−6^ mm^2^/sec, 822 ± 176 × 10^−6^ mm^2^ vs. 943 ± 153 × 10^−6^ mm^2^, 951 ± 211 × 10^−6^ mm^2^/sec vs. 1091 ± 223 × 10^−6^ mm^2^/sec, 1127 ± 227 × 10^−6^ mm^2^ vs. 1282 ± 282 × 10^−6^ mm^2^, and 991 ± 215 × 10^−6^ mm^2^ vs. 1136 ± 211 × 10^−6^ mm^2^ respectively, *p* < 0.05, Table 3). Other ADC variables were not significantly different between ER/PR-positive and -negative groups.

**Table 2:** Comparison of apparent diffusion coefficient (ADC) variables from volume of interest (VOI) of largest brain metastasis according to human epidermal growth receptor 2 (HER2) and estrogen receptor/progesterone receptor (ER/PR) status.

	HER2 status			ER/PR status		
	HER2-negative (n = 16)	HER2- positive (n = 18)	P-value	ER/PR-negative (n = 18)	ER/PR- positive (n = 16)	P-value
Peak location (×10^−6^ mm^2^)	1017 ± 279	945 ± 268	0.45	1026 ± 262	926 ± 281	0.29
25^th^ percentile (×10^−6^ mm^2^)	933 ± 188	883 ± 152	0.39	965 ± 133	840 ± 184	0.03
50^th^ percentile (×10^−6^ mm^2^)	1119 ± 259	1031 ± 215	0.29	1130 ± 229	1007 ± 237	0.13
75^th^ percentile (×10^−6^ mm^2^)	1360 ± 320	1248 ± 295	0.29	1351 ± 316	1244 ± 297	0.31
Mean ADC (×10^−6^ mm^2^)	1167 ± 239	1088 ± 213	0.31	1179 ± 221	1064 ± 222	0.14
Standard deviation (×10^−6^ mm^2^)	327 ± 124	279 ± 113	0.54	291 ± 120	313 ± 120	0.59

**Table 3:** Comparison of apparent diffusion coefficient (ADC) variables from volume of interests (VOI) of multiple brain metastases according to human epidermal growth receptor 2 (HER2) and estrogen receptor/progesterone receptor (ER/PR) status.

	HER2 status			ER/PR status		
	HER2-negative (n = 47)	HER2-positive (n = 38)	P-value	ER/PR-negative (n = 43)	ER/PR-positive (n = 42)	P-value
Peak location(×10^−6^ mm^2^)	974 ± 247	943 ± 274	0.58	1020 ± 275	899 ± 228	0.03
25^th^ percentile (×10^−6^ mm^2^)	890 ± 193	874 ± 150	0.66	943 ± 153	822 ± 176	<0.01
50^th^ percentile (×10^−6^ mm^2^)	1029 ± 242	1012 ± 210	0.73	1091 ± 223	951 ± 211	<0.01
75^th^ percentile (×10^−6^ mm^2^)	1209 ± 308	1201 ± 267	0.91	1282 ± 282	1127 ± 277	0.01
Mean ADC (×10^−6^ mm^2^)	1068 ± 245	1060 ± 198	0.87	1136 ± 211	991 ± 215	<0.01
Standard deviation (×10^−6^ mm^2^)	252 ± 129	256 ± 105	0.87	265.1 ± 108.6	242.7 ± 128.2	0.38

### Prediction of overall survival after brain metastasis and brain metastasis-specific progression-free survival

**Figure 2:**
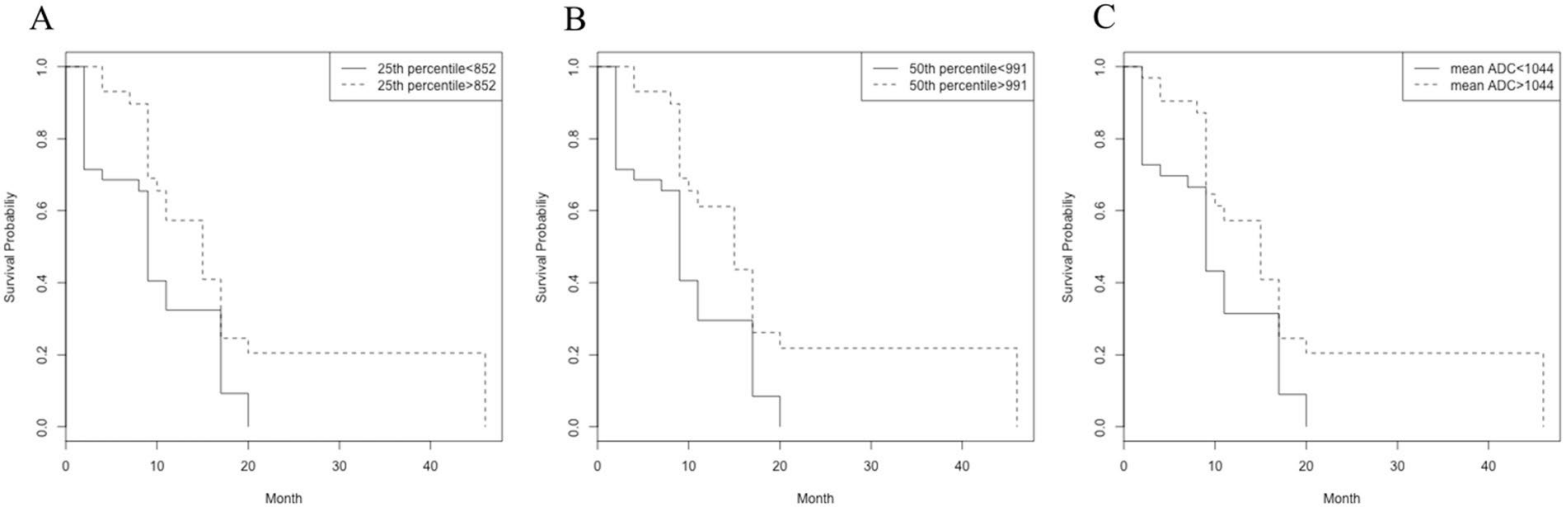
Kaplan-Meier curves for BMPFS show (**A**) 25^th^ percentile ADC (**B**) 50^th^ percentile ADC (**C**) mean ADC. 25^th^ percentile, 50% percentile and mean of ADC significantly stratified BMPFS in breast cancer patients, using ROI method from all measurable BMs.

On univariate analysis with ROI of largest BM, ADC variables were not significant prognostic factors for OSBM and BMPFS. However, with ROIs from all measurable BMs, peak location, 25th percentile, 50th percentile, and mean of ADC histogram were significant prognostic factors for BMPFS but not for OSBM, p < 0.05, Table 4). Lower ADC variables showed poor BMPFS (Fig. 2).

**Table 4:** Survivals in breast cancers with brain metastases depending on apparent diffusion coefficient (ADC) histogram analysis.

Variables from ADC histogram	ROIs with largest BMs		ROIs with all measurable BMs	
	p-value for OSBM	p-value for BMPFS	p-value for OSBM	p-value for BMPFS
Peak location	0.58	0.71	0.41	0.08
25^th^ percentile	0.73	0.31	0.66	0.02
50^th^ percentile	0.38	0.21	0.41	<0.01
75^th^ percentile	0.06	0.44	0.26	0.06
Mean ADC	0.41	0.18	0.48	0.03
Standard deviation	0.22	0.16	0.13	0.24

BMPFS, Brain metastasis-specific progression-free survival; OSBM, overall survival analysis after brain metastasis.

## Discussion

In this study, we tested the hypothesis that ADC histogram analysis of BMs can accurately predict the biological subtypes of breast cancer, and patients’ outcomes. Our results indicated that ER/PR-positive patients have significantly lower 25th percentile of ADC values in BM, compared with ER/PR-negative patients, regardless of ROI methods (ROIs from largest BMs or ROIs from all measurable BMs). Using ROIs from all measurable BMs, peak location, 50th percentile, 75th percentile and mean of ADC were also significantly lower in ER/PR-positive patients than in ER/PR negative-patients. However, ADC variables are not correlated with HER2. Using ROIs from all measurable BMs, peak location, 25th percentile, 50th percentile, and mean of ADC significantly predicted BMPFS, thus they could be a potential prognostic biomarker for BMPFS.

A few previous studies have demonstrated a relationship between DWI and BMs. Hayashida *et al*. evaluated 26 brain metastatic lesions, reporting that small- and large-cell neuroendocrine carcinomas exhibited high signal intensity on DWI. However, their primary lesions generally consisted of lung cancer, and included only one case of primary breast cancer^16^. Duygulu *et al*. studied 87 patients, but concluded that DWI is not correlated with primary tumor histopathology; although their report included 20 breast carcinomas, they did not perform a subgroup analysis^19^. Jung *et al*. limited their cohort to patients with BMs from primary lung cancer; their results demonstrated that ADC values from DWI are significantly correlated with EGFR mutation status, rather than with histology^20^. However, to date, no research has examined the relationship between ADC values and biological features of BMs from primary breast cancer. In the current study, all ADC variables in BMs from breast cancer revealed a decreasing trend in the ER/PR-positive group, compared with the ER/PR-negative group. This result is consistent with previous results in which the median ADC values of primary breast cancer were significantly lower in the ER-positive group than in the ER-negative group^21,22^. This phenomenon can be explained as follows: the ADC value is affected by the molecular diffusion of water, as well as by perfusion^23,24^. Studies using experimental models have shown that ERs inhibit the angiogenic pathway and induce a decrease in perfusion, thus affecting the ADC value^25^. Another noticeable thing in our results is that 25th percentile of ADC shows a significant difference between ER/PR- positive and negative groups regardless of ROI methods. There is a dilemma in choosing ROI method: Using a single representative BM or all measurable BMs from one patient. Either way has flaws, in case of first method, it is hard to select the representative lesion. For the second method, an oversampling issue is raised. However, 25^th^ percentile of ADC is not dependent on ROI method and might be useful to differentiate two groups.

Our results showed that ADC variables are not correlated with the HER2 status of BMs from primary breast cancer. There has been a controversy regarding this issue in primary breast cancer. Jeh *et al*. showed that ADC variables of primary breast cancer were significantly lower in the HER2-positive group, compared with the HER2-negative group^26^. In contrast, Kim *et al*. demonstrated that ADC values are not correlated with HER2 status^21^. Notably, HER2 overexpression induces angiogenesis, thus increasing ADC values; however, this overexpression also stimulates cell proliferation, thereby decreasing ADC values. Thus, we presume that these contradictory effects of HER2 overexpression on ADC values may result in a non-significant relationship between HER2 status and ADC values of BMs in breast cancer.

Some studies have postulated a prognostic value for ADC values in primary breast cancer. Nakajo *et al*. examined 44 breast cancers, and concluded that a low ADC value is significantly correlated with poor prognosis^27^. Mori *et al*. investigated 86 patients with luminal type breast cancer and showed that ADC values are correlated with the Ki-67 labeling index, which is a significant prognostic factor^28^. For BMs, Lee *et al*. evaluated the effect of stereotactic radiosurgery on BMs with ADC maps; they found that increased ADC values are indicators of good tumor control. However, because their primary tumor origins were heterogenous, it is difficult to apply their results to BMs that originate from primary breast cancer. In our study, 25^th^ percentile, 50^th^ percentile, and mean of ADC show potentials to predict BMPFS, specifically using ROIs from all measurable BMs.

Our study has a limitation, the number of our cohort is small for multivariate analysis. We could not verified our independent predictability of our ADC variables. However, our cohort is rather homogenous because patients had been recruited in same institution for a long period (7 years). Also, clinical characteristics between two groups (HER2 positive vs negative, ER/PR positive vs negative) are not different. Thus, our results may serve as a cornerstone for future studies with a larger population to validate and extend these results.

In conclusion, we demonstrated that ADC variables of BMs in breast cancer are significantly lower in ER/PR-positive patients than in ER/PR-negative patients. Specifically, the 25th percentile ADC value are consistently different between two groups regardless of ROI methods. Also, ADC variables of BMs may be a prognostic indicator for BMPFS of breast cancer but these are necessary to be verified in future study with large cohort.

### Data availability

All data generated or analyzed during this study are included in this published article and its Supplementary Information files.

## Electronic supplementary material

Supplemental figure S1

Dataset 1
