# Author Correction: Apparent diffusion coefficient histogram in breast cancer brain metastases may predict their biological subtype and progression

**DOI:** 10.1038/s41598-018-31010-7

**Published:** 2018-08-21

**Authors:** Sung Jun Ahn, Mijin Park, Sungkyu Bang, Eunseo Cho, Sung Gwe Ahn, Sang Hyun Suh, Jong-Min Lee

**Affiliations:** 1 Department of Radiology, Gangnam Severance Hospital, Yonsei University, College of Medicine, Seoul, Korea; 2 Department of Biomedical Engineering, Hanyang University, Seoul, Korea; 3 Department of Surgery, Gangnam Severance Hospital, Yonsei University, College of Medicine, Seoul, Korea

Correction to: *Scientific Reports* 10.1038/s41598-018-28315-y, published online 02 July 2018

In the original version of this Article Jong-Min Lee was incorrectly affiliated with ‘Department of Radiology, Gangnam Severance Hospital, Yonsei University, College of Medicine, Seoul, Korea’. The correct affiliation is listed below.

Department of Biomedical Engineering, Hanyang University, Seoul, Korea.

This error has now been corrected in the PDF and HTML versions of the Article, and in the accompanying Supplementary Information File.

