# Supplementary material for: Apparent diffusion coefficient histogram in breast cancer brain metastases may predict their biological subtype and progression
Source: Sci Rep. 2018 Jul 2;8:9947. doi: 10.1038/s41598-018-28315-y (PMC6028481; doi:10.1038/s41598-018-28315-y)

**Scientific Reports**

**Supplementary information**

**Apparent diffusion coefficient histogram in breast cancer brain metastases may predict their biological subtype and progression**

**Sung Jun Ahn MD^1^, Mijin Park, BS^2^, Sungkyu Bang, BS^2^, Eunseo Cho, BS^2^, Sung Gwe Ahn, MD^3^, Sang Hyun Suh, MD^1^, Jong-Min Lee, PhD^2^**

Department of Radiology^1^ and Surgery^3^, Gangnam Severance Hospital, Yonsei University, College of Medicine, Seoul, Korea

Department of Biomedical Engineering^2^, Hanyang University, Seoul, Korea

Correspondence and requests for materials should be addressed to J.M.L(ljm@hanyang.ac.kr)

This supplementary Information contains one figure.

Supplemental Figure S1. Low ADC values show clustered distribution (red spot) within tumor (blue) in sagittal, coronal and axial images of ADC map.


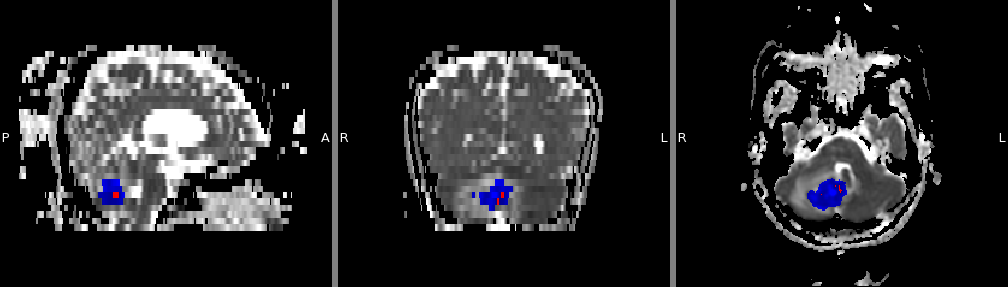

Supplement: Supplementary file 1 — Supplemental figure S1 [file 41598_2018_28315_MOESM1_ESM.docx]
